# Multiplexity and Disruption Propagation in Global Container Liner Shipping Networks: From the Perspective of Carriers’ Geopolitical Affiliations

**DOI:** 10.3390/e28070723

**Published:** 2026-06-24

**Authors:** Huanyu Ren, Xiaozhen Lian, Qiong Chen, Ziheng Lin, Zonghui Jiang, Zhenglong Li

**Affiliations:** 1 Navigation College, Jimei University, Xiamen 361021, China; huanyu_ren@jmu.edu.cn (H.R.); xzhen_l@163.com (X.L.); qchen@jmu.edu.cn (Q.C.); 202421002077@jmu.edu.cn (Z.L.); 202522002082@jmu.edu.cn (Z.J.); 2 School of Civil and Environmental Engineering, Nanyang Technological University, 50 Nanyang Avenue, Singapore 639798, Singapore; 3 School of Economics and Management, Beijing Jiaotong University, Beijing 100044, China

**Keywords:** global container liner shipping networks, geopolitical affiliations, multiplex networks, structural analysis, disruption propagation

## Abstract

Global container liner shipping networks (GCLSNs) underpin world trade, yet their organization is increasingly reshaped by geopolitical fragmentation. Existing studies often model GCLSNs as single-layer networks, overlooking how carriers’ geopolitical affiliations structure both connectivity and disruption risk. This study constructs a weighted carrier–geopolitical multiplex network in which layers are defined by carriers’ geopolitical affiliations and coupled through shared port calls. Structural analysis reveals pronounced asymmetry in layer size, cohesion, and inter-layer dependence, with overlap concentrated in a limited set of shared hubs. Using the Red Sea crisis as an empirical stress-test scenario, we develop a load–capacity propagation model, incorporating intra-layer load redistribution, rerouting to substitute shared hubs, and inter-layer resource squeeze at same-port layer copies. Results show that direct losses concentrate in corridor-exposed layers, while indirect losses propagate selectively through bridge hubs, especially Singapore, Shanghai, Shenzhen, and Port Klang. Sensitivity analysis indicates nonlinear amplification when low tolerance, strong inter-layer squeeze, and elevated rerouting pressure coincide. These findings show that multiplexity does not imply resilience by itself; cross-layer connectivity buffers disruption only when spare capacity is distributed but amplifies vulnerability when it converges on a narrow set of shared hubs. The paper contributes a carrier–geopolitical perspective to shipping network analysis and a dynamic framework for studying disruption propagation in complex logistics systems.

## 1. Introduction

Global container liner shipping networks (GCLSNs) are the circulatory system of world trade, yet their organization is increasingly shaped by geopolitical rivalry, sanctions, alliance restructuring, and strategic state support [1,2,3]. Recent events, including the Red Sea crisis and port-related regulatory initiatives in the United States, show that carriers with different geopolitical affiliations face unequal rerouting pressure, corridor uncertainty, regulatory constraint, and access risk [4,5]. This raises a central question: how does the geopolitical organization of carrier networks shape vulnerability and disruption propagation in the contemporary liner shipping system?

Existing studies have advanced our understanding of maritime connectivity, hub structure, disruption propagation, and transport resilience, but most still aggregate carriers into a single-layer network or interpret disruption mainly through the territorial geography of ports [4,6,7]. This approach overlooks a key structural feature of global container shipping: connectivity is jointly produced by carriers embedded in different geopolitical affiliations, and these carrier-affiliated subnetworks are neither structurally identical nor mutually independent [8,9]. This omission matters because geopolitical affiliation can shape market access, alliance behavior, route design, and dependence on shared ports and corridors.

This study adopts a carrier–geopolitical multiplex perspective and addresses three questions. First, how do carrier–geopolitical layers differ in their internal structure, and through which ports is the multiplex articulated? Second, to what extent do intra-layer cohesion and inter-layer dependence coincide or diverge? Third, how do disruption shocks propagate through shared hubs and cross-layer couplings, and under what conditions do they amplify carrier vulnerability rather than provide redundancy?

To answer these questions, we conceptualize GCLSNs as weighted carrier–geopolitical multiplex networks. Each carrier is assigned to a geopolitical layer following Cohen’s regionalization and the location of its headquarters and principal strategic registration [10]. The resulting network allows the same port to appear in multiple layers while preserving carrier–geopolitical separation in observed route structures. Empirically, we reconstruct yearly networks for 2023–2025 from Clarksons vessel data and AIS-derived voyage records. The structural analysis evaluates layer density, clustering, centralization, efficiency, cohesion, and inter-layer dependence. The propagation analysis develops a load–capacity model for Red Sea disruption scenarios, incorporating intra-layer load redistribution, rerouting to substitute shared hubs, and inter-layer resource squeeze at same-port layer copies.

Building on these gaps, this study advances liner shipping network research in three respects. First, it treats carriers’ geopolitical affiliations as an organizational layer of the shipping network, allowing the same physical port to be represented as multiple port-layer copies when it is served by carriers embedded in different geopolitical blocs. This representation makes geopolitical organization an internal dimension of the network architecture. Second, it separates intra-layer cohesion from inter-layer dependence. This distinction explains why a carrier–geopolitical layer may be internally compact while remaining weakly exposed to the wider multiplex or externally embedded while relying on a concentrated set of shared hubs. Third, it links structural differentiation to disruption propagation through a load–capacity stress-test that records both direct corridor losses and indirect cross-layer losses through shared hubs. The resulting framework identifies propagation interfaces, such as Singapore, Shanghai, Shenzhen, and Port Klang, whose importance depends on their role in connecting directly exposed layers with indirectly affected layers under rerouting pressure.

The remainder of the paper is organized as follows. Section 2 reviews the relevant literature. Section 3 presents the data, structural indicators, and disruption propagation model. Section 4 reports the empirical results. Section 5 discusses the theoretical and analytical implications. Section 6 concludes with implications, limitations, and future research directions.

## 2. Literature Review

### 2.1. Liner Shipping Network Structure and Vulnerability

Research on liner shipping networks has increasingly moved beyond static topology toward disruption-sensitive assessments of vulnerability [7,11], resilience, and connectivity loss [12,13]. Studies of major disruptions show that shocks such as the Suez Canal blockage can reduce global accessibility and connectivity unevenly across regions, vessel types, and trade corridors [14]. Related work further argues that vulnerability assessment should account for port irreplaceability [15], hinterland relations, spatial agglomeration, and geopolitical environments, since route substitution is constrained by both geography and operational capacity rather than topology alone [16]. Cascading-failure approaches extend this perspective by emphasizing load redistribution, critical thresholds, and node importance under extreme events and regional conflicts [2].

The above literature establishes that liner shipping networks are exposed to both localized and systemic disruptions. However, most studies still represent the system as an aggregated graph of ports and routes. Such representations are useful for identifying critical ports, but they suppress the organizational heterogeneity behind observed connectivity. In practice, shipping links are produced by carriers with different market positions, alliance relations, ownership structures, and geopolitical affiliations. Treating these carriers as a homogeneous background limits our ability to explain why similar topological positions may imply different exposure, dependence, or propagation consequences. A related transport-resilience study also stresses that global mobility systems should be analyzed as interconnected infrastructures rather than isolated modal networks [17]. This view is highly relevant to liner shipping; resilience depends on the interaction between network structure, disruption mechanism, and adaptive response. Yet the carrier organization that generates liner connectivity remains weakly integrated into network models. Carrier heterogeneity is often acknowledged descriptively but rarely formalized as a source of layered network structure.

### 2.2. Geopolitical Exposure, Carrier Organization, and Multiplex Dependence

Recent maritime studies show that geopolitical instability increasingly reshapes shipping connectivity. The Russia–Ukraine war, the Red Sea crisis, and related regional disruptions have altered maritime trade flows, increased logistics costs [18], changed port competitiveness [19], and triggered persistent foreland and route restructuring [20,21,22]. These studies demonstrate that geopolitical shocks affect liner shipping not only by blocking specific corridors but also by reorganizing the roles of ports, regions, and service networks.

Carrier-focused studies provide a complementary but still separate perspective. Carrier presence influences port pricing, profitability, and competitive outcomes [23], while industry consolidation and mergers reflect spatial, strategic, and organizational logics [24]. Broader supply-chain research further suggests that geopolitical disruptions require redesign, regionalization, transparency, and resilience-oriented adaptation [25]. These insights confirm that carriers are not passive users of a transport network; they actively shape connectivity, competition, and adaptation.

The problem is that these two bodies of literature remain weakly connected. Geopolitical studies tend to focus on disrupted regions, corridors, or events, whereas carrier studies usually focus on market conduct, concentration, and firm restructuring. What remains underdeveloped is a framework that links geopolitical exposure to the carrier-generated structure of the global liner network. This is precisely where a multiplex perspective becomes useful. If layers are defined by carriers’ geopolitical affiliations, then the same port can be embedded in multiple organizational subnetworks, and inter-layer dependence can be measured through shared ports, overlapping routes, and bridge hubs. Such a representation allows geopolitical affiliation to enter the network architecture itself rather than being treated as an external explanatory variable.

### 2.3. Disruption Propagation in Complex and Interdependent Networks

The broader complex-network literature provides the theoretical basis for analyzing disruption propagation in such a multiplex system. Classic studies show that complex networks are often robust to random failures but fragile to targeted attacks on critical nodes [26,27,28]. In flow-based networks, node or link failures can trigger load redistribution and cascading overloads when flows concentrate on a limited number of hubs [29,30,31]. Recent work further shows that cascading risk is shaped by capacity allocation, recovery thresholds, delayed interactions, and dependency links across interdependent networks [32,33,34].

This logic maps directly onto carrier–geopolitical liner networks. Disruption may spread within a carrier–geopolitical layer through route dependencies and load redistribution while also spilling across layers through shared ports and overlapping services [35,36,37]. Cross-layer dependence can therefore have two opposing effects. It may provide redundancy by allowing rerouting and substitute connectivity, but it may also concentrate pressure at shared hubs and convert local disruption into cross-layer vulnerability. Whether multiplexity buffers or amplifies disruption depends on load, spare capacity, rerouting pressure, and the strength of inter-layer coupling. Despite this relevance, disruption propagation in liner shipping is still rarely modeled as a carrier–geopolitical multiplex process. Existing cascading-failure studies usually operate on aggregate networks, while geopolitical disruption studies often remain scenario-specific. As a result, they have limited capacity to explain how carrier affiliation, shared hubs, and cross-layer coupling jointly shape propagation paths and nonlinear amplification.

### 2.4. Research Gaps and Contributions

Three gaps follow from the literature. First, liner shipping vulnerability studies have improved the measurement of connectivity degradation, port resilience, and cascading failure, but they still mainly analyze the system as a single aggregated network. This limits their ability to identify the organizational sources of systemic exposure before disruption occurs. Second, geopolitical studies show that wars, regional conflicts, and geoeconomic shocks reconfigure maritime networks, but they rarely explain how geopolitical exposure is embedded in global liner connectivity through carrier groups operating across multiple ports and routes. Third, carrier studies demonstrate that firm organization and market structure matter, but they seldom translate carrier heterogeneity into a multiplex representation suitable for network vulnerability and propagation analysis.

This study addresses these gaps by modelling GCLSNs as weighted carrier–geopolitical multiplex networks. Layers are defined by carriers’ geopolitical affiliations, and inter-layer dependence is measured through shared ports, overlapping routes, and bridge hubs. We further link structural diagnostics with a load–capacity propagation model that captures intra-layer load redistribution, rerouting to substitute shared hubs, and inter-layer resource squeeze. This framework explains not only where vulnerabilities are located but also how disruption propagates across carrier–geopolitical layers under geopolitical stress.

This design connects three strands of prior research that have often developed separately. Studies of multiplex shipping networks have shown that carrier- or service-based layers reveal overlap patterns hidden in aggregate graphs. Studies of cascading failure and transport resilience have explained how load redistribution can amplify disruption in interdependent networks. Studies of maritime geopolitical disruption have documented how corridor crises, including the Red Sea crisis, reshape port calls, routes, and regional connectivity. The present framework brings these strands together by asking how carrier-affiliated geopolitical layers shape the conversion of a corridor shock into selective cross-layer propagation. In this sense, ports are analyzed not only as territorial transport nodes but also as interfaces among organizationally differentiated carrier layers.

## 3. Methodology

### 3.1. Data Collection and Network Construction

#### 3.1.1. Data Collection

This study constructs a carrier-centered liner shipping dataset through three linked steps: carrier selection, vessel identification, and AIS-based movement reconstruction. First, major liner carriers are identified from the Clarksons database, and carriers representing approximately 80% of the global liner market are retained. This threshold balances representativeness and data tractability while capturing the dominant organizational structure of global container shipping. Second, vessel-level information is collected for the selected carriers, including ship identity, technical attributes, and MMSI. MMSI is used as the unique key linking carrier–vessel records with dynamic AIS trajectories. Third, AIS traces are matched by MMSI and converted into time-stamped port-call sequences. Consecutive port calls are then transformed into directed origin–destination voyage segments, which form the empirical basis for network construction.

Terminal-level and sub-port labels are standardized into canonical port–complex nodes before network construction, reducing artificial node fragmentation. The final observation window covers 2023–2025 and contains 559,481 observed port-to-port voyage segments from 27 carriers. The annual networks include 991 ports and 10,993 directed routes in 2023, 1001 ports and 13,226 routes in 2024, and 897 ports and 14,574 routes in 2025. This pattern indicates a denser but more selective connectivity structure, motivating the analysis of multiplexity and disruption propagation under geopolitical segmentation.

#### 3.1.2. Multiplex Network Construction

Carrier–geopolitical affiliation is defined at the firm level. Following Cohen’s geopolitical regionalization [10], each liner carrier is assigned to a geopolitical region according to its headquarters and principal strategic registration. All vessels operated by the carrier inherit the same affiliation, and each AIS-derived voyage segment is assigned to a geopolitical layer according to the affiliation of the operating carrier. This design treats geopolitical affiliation as an organizational attribute of carriers rather than a territorial attribute of ports. The geopolitical affiliations of carriers in this study are presented in Appendix A Table A1.

This geopolitical layer should be interpreted as a strategic affiliation layer rather than as a complete operational community. It does not imply that carriers within the same geopolitical region behave uniformly, nor that headquarters location alone determines service decisions. Carrier behavior may also be shaped by alliance membership, vessel-sharing agreements, subsidiaries, ownership links, chartering arrangements, service cooperation, and port-call strategies. We therefore use the carrier–geopolitical layer to examine whether broad carrier-affiliation blocs are associated with differentiated network positions and propagation pathways, and we evaluate this design through benchmark checks against aggregate port structure, carrier-level port overlap, and randomized carrier-to-layer assignments.

Based on AIS-derived voyage records, we first construct yearly directed and weighted liner shipping networks. Ports are nodes, and consecutive port calls are directed edges. If a vessel departs from port i and subsequently arrives at port j in year t, a directed link from i to j is created. Edge weight records the number of observed voyages of that port pair within the year.

To incorporate carriers’ geopolitical affiliations, the global network is decomposed into multiple geopolitical layers. For each year t, voyages are assigned to layer l according to the geopolitical affiliation of the operating carrier. Thus, each layer represents the port-to-port service network operated by carriers affiliated with a specific geopolitical region. Formally, the layer-specific network is defined as:

(1) $$G_{t}^{\left(l\right)}=(V_{t}^{\left(l\right)},E_{t}^{\left(l\right)},W_{t}^{\left(l\right)}),$$

where $V_{t}^{\left(l\right)}$ is the set of ports served by carriers in geopolitical region l during year t, $E_{t}^{\left(l\right)}$ is the set of directed port-to-port links operated by those carriers, and $W_{t}^{\left(l\right)}$ is the corresponding edge-weight matrix. The weight of the edge from i to j in layer l is:

(2) $$w_{ij,t}^{\left(l\right)}=\sum_{r\in R_{t}}I(o_{r}=i,d_{r}=j,g_{r}=l),$$

where $R_{t}$ is the set of observed voyage segments in year t, $o_{r}$ and $d_{r}$ denote the origin and destination ports of segment r, $g_{r}$ is the geopolitical affiliation of the operating carrier, and I(⋅) is an indicator function.

The complete multiplex network is then represented as:

(3) $$M_{t}=\{{G_{t}}^{\left(l\right)}:l\in L\},C_{t},$$

where L denotes the set of geopolitical regions and $C_{t}$ denotes inter-layer couplings. Inter-layer couplings are defined through shared ports. If the same physical port appears in two layers, an identity link is created between its layer-specific copies:

(4) $$c_{i,t}^{\left(l,h\right)}=\left\{\begin{array}{l} 1,\;i\in V_{t}^{\left(l\right)}\cap V_{t}^{\left(h\right)},\;l\neq h,\\ 0,\;\mathrm{otherwise} \end{array}\right.$$

**Figure 1:**
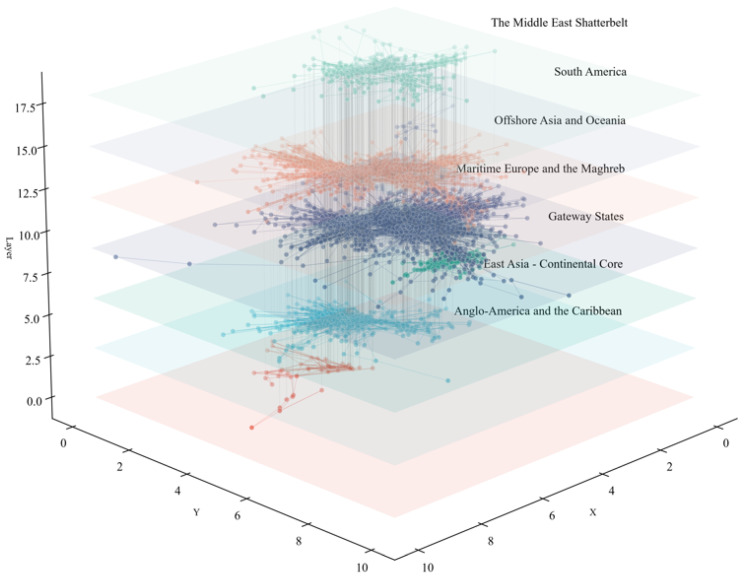
Schematic representation of the carrier–geopolitical multiplex network.

In this framework, intra-layer edges are directed and weighted by voyage frequency, while inter-layer links are binary identity couplings based on shared ports (see Figure 1). The resulting multiplex network captures how carrier-affiliated geopolitical subnetworks are both separated by corporate geopolitical affiliation and interconnected through common maritime infrastructures.

In Figure 1, each dot represents a port-layer copy rather than a different physical port. For example, the same port may appear as separate dots in Maritime Europe, Offshore Asia, or East Asia Core if carriers from those geopolitical affiliations call at that port. Horizontal or within-layer links denote observed directed port-to-port voyage movements, whereas inter-layer identity links denote shared physical ports across carrier–geopolitical layers. This interpretation is made explicit in the figure caption and in the supplementary interactive visualization (Figure S1).

### 3.2. Structural Indicators

The structural analysis evaluates both internal layer organization and cross-layer dependence. For each layer l in year t, basic scale is measured by the number of ports $n_{l,t}$ and directed routes $m_{l,t}$. Directed density is defined as:

(5) $$\rho_{l,t}=\frac{m_{l,t}}{n_{l,t}(n_{l,t}-1)}.$$

We further calculate the average clustering coefficient, network efficiency, and degree centralization. Efficiency is measured as:

(6) $$Eff_{l,t}=\frac{1}{n_{l,t}(n_{l,t}-1)}\sum_{i\neq j}\frac{1}{d_{ij}^{\left(l,t\right)}},$$

where $\;d_{ij}^{\left(l,t\right)}$ is the directed shortest-path distance between ports i and j in layer l.

Centralization is measured as the mean of the in-degree and out-degree Gini coefficients. For layer α, let $k_{i}^{\alpha }$ denote the degree of port i, and let $n^{\alpha }=\left|V^{\alpha }\right|$ be the number of ports in the layer. After arranging port degrees in non-decreasing order, $k_{\left(1\right)}^{\alpha }\leq k_{\left(2\right)}^{\alpha }\leq \ldots \leq k_{\left(n\right)}^{\alpha }$, the degree Gini coefficient is calculated as: $G^{\alpha }=\frac{2\sum_{i=1}^{n^{\alpha }}ik_{\left(i\right)}^{\alpha }}{n^{\alpha }\sum_{i=1}^{n^{\alpha }}k_{\left(i\right)}^{\alpha }}-\frac{n^{\alpha }+1}{n^{\alpha }}$, where $G^{\alpha }$ ranges from 0 to 1. A value close to 0 indicates that connections are relatively evenly distributed among ports, while a value close to 1 indicates that connectivity is highly concentrated in a small number of hub ports. For a directed layer l in year t, the centralization is calculated as:

(7) $$Cent_{l,t}=\frac{Gini_{l,t}^{in}+Gini_{l,t}^{out}}{2}.$$

To summarize intra-layer organization, we construct a composite cohesion index:

(8) $$Cohesion_{l,t}=\frac{z(\rho_{l,t})+z(Clust_{l,t})+z(Eff_{l,t})-z(Cent_{l,t})}{4},$$

where $Clust_{l,t}$ denotes the average clustering coefficient and z(⋅) denotes standardization. Higher values indicate denser, more clustered, and more efficiently connected layers that are less strongly concentrated in a small number of hubs.

Multiplexity is evaluated by layer count and the multiplex participation coefficient:

(9) $$MPC_{i}=1-\sum_{l}{\left(\frac{k_{i}^{\left(l\right)}}{\sum_{h}k_{i}^{\left(h\right)}}\right)}^{2},$$

where $k_{i}^{\left(l\right)}$ is the degree of port i in layer l. Higher values indicate more balanced participation across layers.

Inter-layer dependence is evaluated with three pairwise indicators: Port overlap, measured by the Jaccard index of port sets:

(10) $$PO_{lh}=\frac{\left|V^{\left(l\right)}\cap V^{\left(h\right)}\right|}{\left|V^{\left(l\right)}\cup V^{\left(h\right)}\right|}$$

Route overlap, measured by the Jaccard index of directed route sets:

(11) $$RO_{lh}=\frac{\left|E^{l}\cap E^{h}\right|}{\left|E^{l}\cup E^{h}\right|}$$

Degree-profile similarity, measured by the Pearson correlation of port degree profiles across two layers:

(12) $$DS_{lh}=corr(k_{i}^{l},k_{i}^{h}),\;i\in V^{\left(l\right)}\cup V^{\left(h\right)}.$$

### 3.3. Disruption Propagation Model

In this section, disruption is modeled as a load–capacity process on the carrier–geopolitical multiplex. The model is used as a comparative stress-test framework rather than as a calibrated engineering forecast of port throughput, berth occupancy, yard utilization, vessel schedule slack, or transshipment capacity. Its purpose is to evaluate how disruption pressure can be redistributed within and across carrier–geopolitical layers under controlled parameter settings. Each port-layer copy is treated as a state node (i,l), where i denotes a physical port and l denotes a geopolitical–carrier layer. Its initial operating load is denoted by $L_{i,l}(0)$ and is proxied by the normalized weighted betweenness of port i in layer l. Weighted betweenness is therefore interpreted as a network-load proxy, not as a direct measurement of physical port workload. It captures the brokerage role of a port-layer copy in the observed directed service network: ports lying on more weighted shortest paths are more likely to mediate route continuity and rerouting pressure when corridor disruption occurs. Node capacity is specified as a nonlinear function of pre-shock load:

(13) $$C_{i,l}=L_{i,l}(0)+\alpha_{l}{\left[L_{i,l}(0)\right]}^{\eta },$$

where $\alpha_{l}$ is the layer-specific tolerance coefficient and η controls how reserve capacity scales with node importance. A state node (i,l) becomes overloaded whenever its realized load ($L_{i,l}(t)$) exceeds its effective capacity ($C_{i,l}$). The capacity function should be read as a stylized reserve-capacity function around pre-shock network load. The tolerance coefficient controls the spare margin above baseline load, while the nonlinearity parameter allows reserve capacity to scale differently for low- and high-load ports. These parameters are stress-test controls rather than empirically measured engineering capacities.

The first transmission channel is intra-layer cascading, which represents congestion propagation within the physical service network. When state node (i,l) becomes overloaded, its excess load is:

(14) $$O_{i,l}(t)=\max\{L_{i,l}(t)-C_{i,l}^{eff}(t),0\}$$

Only a share (1−r) of this excess load is redistributed to the active neighbors of i within the same layer l according to:

(15) $$\Delta L_{j\leftarrow i}^{intra}(t)=(1-r)O_{i,l}(t)\frac{{\tilde{w}}_{ij}^{\left(l\right)}{\left[RC_{j,l}(t)\right]}^{\mu }}{\sum_{k\in N_{i,l}^{A}}{\tilde{w}}_{ik}^{\left(l\right)}{\left[RC_{k,l}(t)\right]}^{\mu }}$$

where ${\tilde{w}}_{ij}^{\left(l\right)}$ is the normalized route intensity from port i to neighbor j in layer l, $N_{i,l}^{A}$ is the set of non-failed neighbors of port i within layer l, $RC_{j,l}(t)=\max\{C_{j,l}^{eff}(t)-L_{j,l}(t),0\}$ is the residual capacity of neighbor j, and μ controls whether redistribution favors topologically strong neighbors or neighbors with larger spare capacity.

The remaining share $rO_{i,l}(t)$ is treated as detoured traffic and reassigned to substitute shared hubs within the same layer:

(16) $$\Delta L_{h\leftarrow i}^{reroute}(t)=rO_{i,l}(t)\frac{\psi_{h,l}{\left(1+d_{ih}^{\left(l\right)}\right)}^{-1}}{\sum_{m\in H_{i,l}}\psi_{m,l}{\left(1+d_{im}^{\left(l\right)}\right)}^{-1}},$$

where $H_{i,l}$ is the set of reachable shared hubs for port i in layer l, $d_{ih}^{\left(l\right)}$ is the weighted shortest-path distance from i to candidate hub h, and $\psi_{h,l}$ combines cross-layer hub importance with the hub’s pre-shock load. This second intra-layer rule captures emergency rerouting from disrupted corridor nodes toward substitute transshipment hubs. For directly attacked Red Sea corridor nodes, an additional initial detour demand $sL_{i,l}(0)$ is injected into substitute hubs at t=0 to mimic route diversion around the disrupted corridor.

The second transmission channel is inter-layer spillover, which captures the resource squeeze effect at shared hubs. For a physical port p shared by layers l and h, pressure in one layer reduces the effective capacity available to the others:

(17) $$P_{p,l}(t)=\max\{L_{p,l}(t)-L_{p,l}(0),0\}+\max\{L_{p,l}(t)-C_{p,l},0\},$$

(18) $$C_{p,h}^{eff}(t)=C_{p,h}-\delta \sum_{l\neq h}\phi_{p}\Gamma_{lh}P_{p,l}(t-1),$$

where δ is spillover intensity, $\phi_{p}$ denotes the normalized cross-layer hub importance of shared port p, and $\Gamma_{lh}$ denotes empirical layer coupling derived from observed inter-layer overlap. The pressure term combines excess demand over the node’s pre-shock load with residual overload beyond its baseline capacity, thereby representing how one layer’s surge at a shared hub occupies berth, yard, transshipment, and schedule slack that would otherwise serve other layers.

We use the Red Sea crisis to distinguish direct attack from indirect spillover. Let R denote the Red Sea–Suez corridor node set. Because the multiplex is defined by carriers’ geopolitical affiliations rather than by ports’ territorial identities, direct and indirect effects are identified by corridor exposure rather than by redefining the layers themselves. Operationally, AIS-derived port-call sequences are first converted into directed consecutive port-to-port voyage segments within each carrier–geopolitical layer. A segment is treated as touching the Red Sea–Suez corridor when either its departure port or arrival port belongs to the corridor proxy set. The proxy set includesPort Said, Sokhna, Jeddah, Al Aqaba, Djibouti, Salalah, King Abdullah, Qadimah, Duba, and Damietta.

A layer is classified as directly exposed when at least 2% of its observed 2025 port-to-port segments touch this corridor proxy set. This rule is an observable lower-bound proxy for corridor dependence rather than a complete reconstruction of canal transit or long-distance service rotations. It captures services with explicit Red Sea–Suez corridor port calls, but it may undercount services that transit the canal or are rerouted around the Cape of Good Hope without calling at one of the corridor proxy ports. For this reason, the Red Sea scenario is interpreted as a conservative stress-test scenario rather than a full operational reconstruction of all post-crisis route adjustments.

This AIS-derived port-call rule is a conservative observable proxy for corridor dependence: it captures services with explicit Red Sea–Suez port calls but does not claim to reconstruct every canal transit or long-distance rotation that may have been rerouted during the crisis. We therefore treat the scenario as an empirically grounded stress test and report sensitivity analyses around it. Corridor exposure is measured as:

(19) $$E_{l}^{RS}=\frac{n_{l}^{RS}}{n_{l}},$$

where $n_{l}^{RS}$ is the number of observed voyage segments in layer l whose departure or arrival port belongs to the corridor set, and $n_{l}$ is the total number of observed segments in that layer. Applying this rule to the 2025 AIS-derived segment table identifies four directly exposed layers: Gateway States, the Middle East Shatterbelt, Maritime Europe and the Maghreb, and Offshore Asia and Oceania. Their corridor-exposure shares are 6.89%, 3.77%, 3.22%, and 2.67%, respectively. Anglo-America and the Caribbean, East Asia—Continental Core, and South America have no observed segments touching the corridor proxy set in the 2025 data and are therefore treated as indirectly affected layers if they are connected to attacked layers through shared ports.

Layers with $E_{l}^{RS}\geq \theta$ are treated as directly attacked layers. For corridor nodes in those layers, initial capacity is reduced by shock intensity s:

(20) $$C_{i,l}^{eff}(0)=(1-s)C_{i,l},\;i\in R.$$

Layers with lower corridor exposure but non-zero coupling to attacked layers are treated as indirectly affected layers. This design makes it possible to compare directly hit and indirectly affected layers within the same carrier–geopolitical multiplex.

The model is evaluated through parameterized simulation rather than single-point calibration. Definitions of different parameters are summarized in Table 1.

We vary the tolerance coefficient (α), capacity nonlinearity (η), redistribution preference (μ), rerouting share (r), spillover intensity (δ), and shock intensity (s) across conservative, baseline, amplified, one-at-a-time, and two-dimensional phase-diagram settings. The simulation is then iterated until either a steady state is reached or a fixed horizon (T) is met. To capture operational degradation before complete node failure, performance is computed on a congestion-adjusted graph. Each active node receives a service factor:

(21) $$g_{i,l}(t)=\min\left\{\frac{C_{i,l}^{eff}(t)}{C_{i,l}},1\right\}\times \min\left\{\frac{C_{i,l}^{eff}(t)}{L_{i,l}(t)},1\right\},$$

and each active edge is down-weighted by the minimum service factor of its endpoints. On this basis, the first core impact indicator is the network functional loss rate:

(22) $$NFLR_{l}(t)=1-\frac{\lambda T_{l}(t)+(1-\lambda )Eff_{l}(t)}{\lambda T_{l}(0)+(1-\lambda )Eff_{l}(0)},$$

where $T_{l}(t)$ is the active weighted throughput of layer l, $Eff_{l}(t)$ is post-shock efficiency, and λ∈[0,1] balances throughput and topological performance.

The second is inter-layer transmission efficiency from attacked layer a to target layer b:

(23) $$ITE_{a\to b}=\frac{\sum_{t=1}^{T}NFLR_{b}(t)}{\sum_{t=1}^{T}NFLR_{a}(t)}.$$

Higher ITE values indicate that a larger share of direct damage is converted into indirect loss in other layers.

To reveal risk asymmetry, we compare peak loss, cumulative loss, and time-to-peak between directly attacked layers and indirectly affected layers. Beyond the conservative-baseline-amplified regimes, we also conduct one-at-a-time parameter scans and pairwise phase-diagram analysis in the (α,δ) and (δ,r) spaces in order to identify threshold effects, nonlinear amplification, and the conditions under which bridge-hub squeeze becomes the dominant transmission mechanism. This framework makes it possible to identify not only where disruption starts but also how geopolitical affiliation shapes the direction, intensity, and temporal profile of multiplex risk transmission.

To recover the main transmission routes of secondary disruption, the simulation also records each inter-layer squeeze event at shared hubs and aggregates these events into path tuples of the form “source layer → bridge port → target layer”. The resulting path shares identify which bridge hubs and which layer pairs carry the largest portion of direct-to-indirect spillover under the baseline scenario.

### 3.4. Benchmark and Robustness Checks for Layer Construction

To evaluate whether the carrier–geopolitical multiplex produces information beyond conventional network representations, we conduct three benchmark and robustness checks for the 2025 network. First, we compare the dominant propagation hubs with endpoint-volume rankings in the aggregated single-layer port network. This benchmark tests whether the reported bridge hubs are simply the largest ports in the global network. Second, we construct a carrier-level overlap benchmark by counting the number of distinct carriers serving each port. This benchmark tests whether the results can be explained by broad multi-carrier presence rather than by layer-specific propagation structure. Third, we perform 500 random carrier-to-layer permutations while preserving the number of carriers assigned to each geopolitical layer and recompute pairwise port-overlap structure. This test evaluates whether the observed geopolitical grouping is distinguishable from random carrier grouping with the same layer sizes.

These checks are designed as bounded robustness tests for the proposed layer construction. They assess whether the main structural and propagation results are reducible to aggregate port size, carrier count, or random grouping. A complete alliance-based, ownership-based, or vessel-sharing-agreement-based multiplex reconstruction would require additional commercial and contractual data, while validation against historical disruption outcomes would require service-level post-crisis route changes and schedule-adjustment records. We therefore treat such comparisons as important extensions, while using the present benchmarks to test whether the strategic-affiliation layer available from the current data adds information beyond conventional representations.

## 4. Results

### 4.1. Structural Differentiation Across Carrier–Geopolitical Layers

Table 2 shows that layer structure does not coincide mechanically. Maritime Europe has the highest efficiency (1.494), but its cohesion index is only moderately positive (0.153) because this reach is supported by a highly centralized structure. Offshore Asia’s cohesion index is negative (−0.219), indicating weaker internal integration relative to hub concentration. East Asia Core occupies an intermediate position, whereas the Middle East combines modest scale with the lowest efficiency among the medium-sized layers (0.764) and has a cohesion index of −0.345. Anglo-America and South America appear highly cohesive, but these values should be interpreted cautiously because small-network effects mechanically inflate density and clustering.

**Figure 2:**
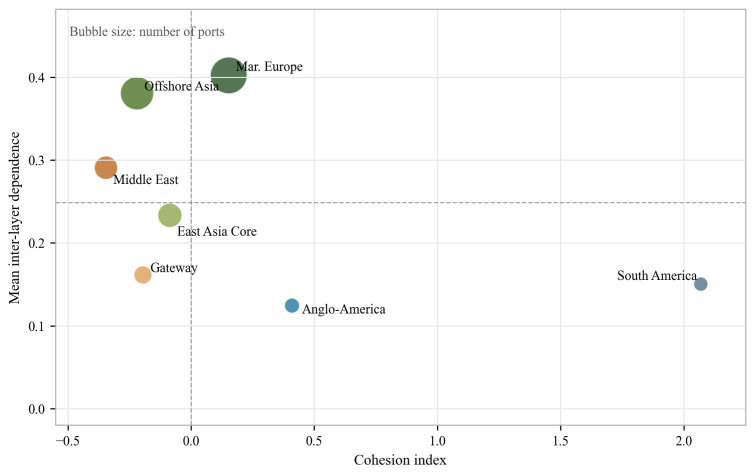
Cohesion–dependence positioning of carrier–geopolitical layers in 2025.

Figure 2 adds a second structural angle by positioning each layer according to internal cohesion and mean inter-layer dependence. Maritime Europe and Offshore Asia are the most externally embedded layers, but only Maritime Europe has positive cohesion. The Middle East combines weak cohesion with relatively high dependence, suggesting a layer that is externally exposed without being internally robust. By contrast, Anglo-America and South America are internally cohesive but externally peripheral. This separation confirms that cohesion and dependence are distinct dimensions rather than different expressions of the same structural property.

**Figure 3:**
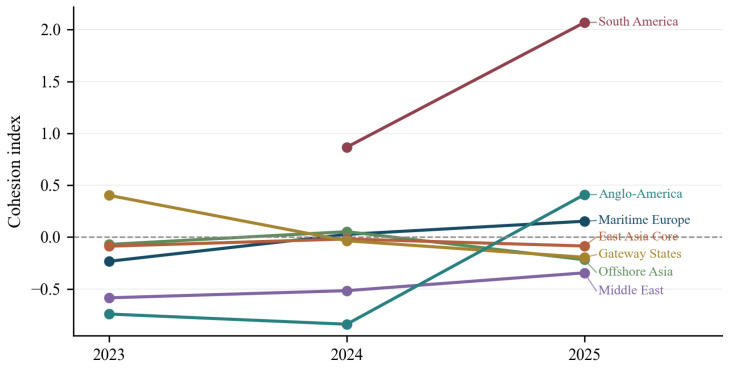
Cohesion-index trajectories by carrier–geopolitical layer.

Figure 3 shows that these differences are persistent but not static. Maritime Europe shifts from negative cohesion in 2023 to positive cohesion in 2025, whereas the Middle East remains below zero throughout. Offshore Asia improves in 2024 and then declines again in 2025, while East Asia Core remains comparatively stable. The first result is therefore that the carrier–geopolitical multiplex is not composed of equivalent layers but of subnetworks that differ sharply in scale, efficiency, centralization, cohesion, and external embeddedness.

### 4.2. Inter-Layer Dependence

**Figure 4:**
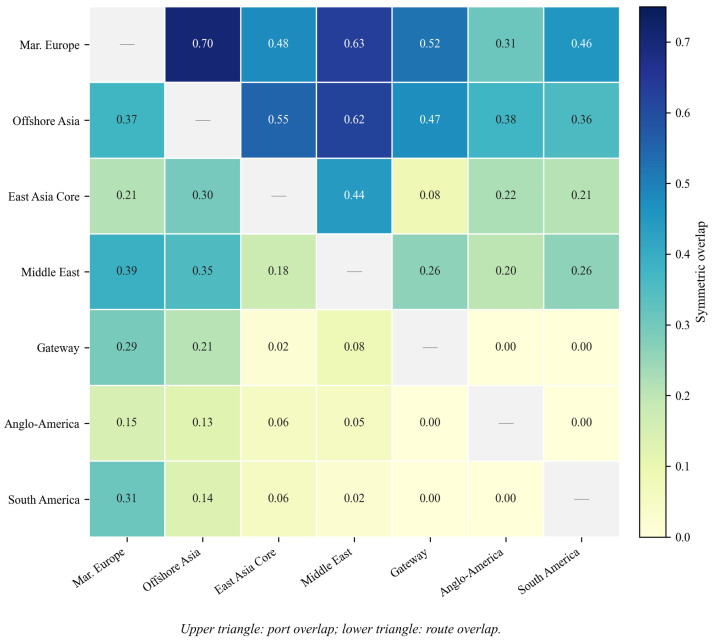
Inter-layer dependence matrix in 2025.

Figure 4 condenses inter-layer dependence into a single matrix. The upper triangle reports symmetric port overlap and the lower triangle symmetric route overlap. A dense dependence core is clearly visible among Maritime Europe, Offshore Asia, the Middle East, and East Asia Core. The strongest pair is Maritime Europe–Offshore Asia, with a port overlap value of 0.70 and a route overlap value of 0.37. Offshore Asia also overlaps strongly with the Middle East (0.62; 0.35) and East Asia Core (0.55; 0.30), while Maritime Europe retains substantial dependence on the Middle East (0.63; 0.39). These values indicate that the main carrier–geopolitical layers are not loosely juxtaposed; they are structurally entangled through repeated sharing of ports and, to a lesser extent, routes.

The matrix also highlights asymmetric embedding at the periphery. The Gateway States show moderate symmetric dependence on Maritime Europe (0.52 port overlap; 0.29 route overlap) and Offshore Asia (0.47; 0.21), but they display much weaker links to the other small layers. The underlying directional coverage ratios are even more revealing: 93.2% of Gateway States ports are covered by Maritime Europe and 81.8% by Offshore Asia. Thus, inter-layer dependence is not only a matter of mutual overlap among large layers; it also reflects the extent to which smaller layers are absorbed into larger carrier–geopolitical structures.

Taken together, the structural results answer the first two research questions in a tightly connected way. Carrier–geopolitical layers differ markedly in their internal organization, yet the largest of them also form the strongest inter-layer dependence core. Internal cohesion and external dependence are therefore related but distinct dimensions; some layers are internally coherent without being strongly embedded, whereas others are extensive and highly interdependent without being especially cohesive.

### 4.3. Propagation of Red Sea Disruption

The propagation analysis focuses on four results: baseline cascade timing, direct–indirect asymmetry, dominant transmission paths, and parameter conditions that convert overlap into spillover. Because the model is not calibrated to predict absolute operational losses, the results are interpreted as mechanism-oriented stress-test outcomes. We therefore focus on qualitative patterns that remain interpretable across parameter regimes, including direct–indirect asymmetry, threshold behavior, and the concentration of spillover through shared bridge hubs.

**Figure 5:**
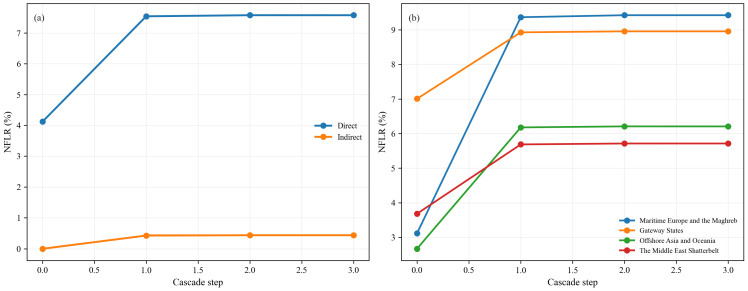
Baseline load–capacity trajectories in the Red Sea disruption scenario. Note: (**a**) network-level direct and indirect NELR across cascade steps; (**b**) layer-specific NELR trajectories for the affected carrier-geopolitical layers.

Figure 5 shows that the Red Sea shock propagates as an asymmetric cascade rather than as an immediate system-wide collapse. Under the baseline parameter regime, the mean network functional loss rate (NFLR) of directly attacked layers peaks at 7.58% by step 2, whereas the indirect group peaks one step later at only 0.43%. The ordering of the most affected layers is also clearly structured. Maritime Europe and the Maghreb and Gateway States form the main direct-loss core, followed by Offshore Asia and Oceania and the Middle East Shatterbelt, while East Asia—Continental Core is the only indirect layer that exhibits a visible secondary response. The temporal lag between the two groups indicates that indirect loss is transmitted through shared hubs and overlapping layer structures rather than being generated simultaneously with the initial corridor shock.

Table 3 formalizes this asymmetry. Among directly attacked layers, the Maritime Europe and the Maghreb layer records the highest peak NFLR (9.4%), while the Gateway States combine the highest corridor exposure (6.9%) with the largest cumulative NFLR (0.34). Offshore Asia and Oceania and the Middle East Shatterbelt experience smaller but still substantial peak losses of 6.2% and 5.7%. Indirect layers remain much less affected: East Asia—Continental Core peaks at 1.0% with an inter-layer transmission efficiency (ITE) of 0.11, Anglo-America and the Caribbean reaches 0.3%, and South America remains close to zero. The effect is therefore strongly asymmetric, but it is not fully contained within the directly exposed layers.

**Figure 6:**
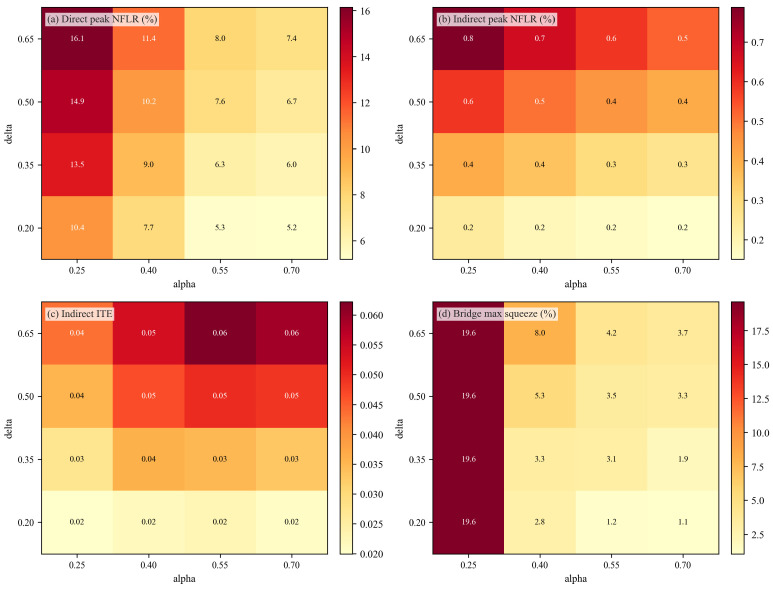
Alpha–delta phase diagrams.

Figure 6 reveals a clear threshold pattern. Indirect losses remain modest across much of the (α,δ) space, but they rise sharply when tolerance is low and spillover intensity is high. The strongest indirect-loss cell occurs at α=0.25 and δ=0.65, where indirect peak NFLR reaches 0.8% and bridge-hub squeeze exceeds 19%. Cross-layer vulnerability is therefore not produced by coupling alone. It emerges when weak reserve capacity and strong inter-layer squeeze coincide, allowing pressure created in directly attacked layers to saturate shared hubs and spill into otherwise non-exposed layers.

**Figure 7:**
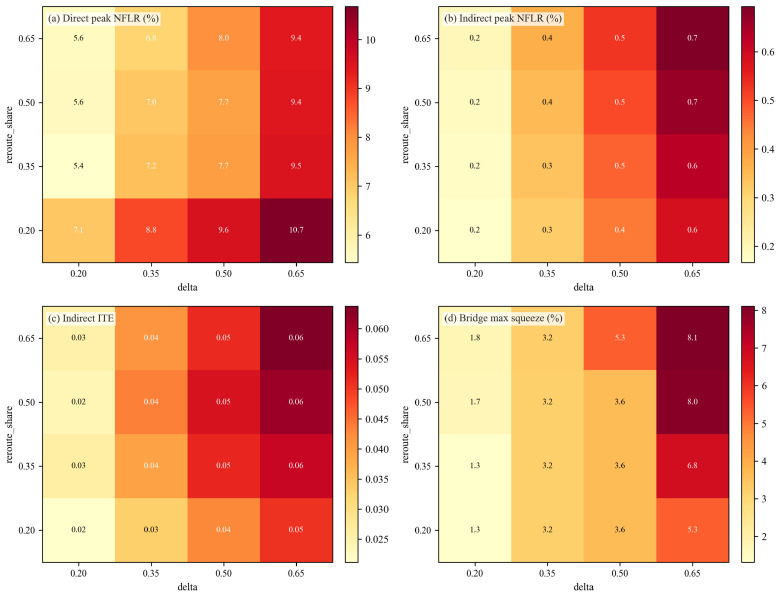
Delta–reroute phase diagrams.

As shown in Figure 7, a complementary (δ,r) scan leads to a more selective interpretation of rerouting. When δ remains low, increasing the rerouting share r generates only limited additional indirect loss. Once δ rises toward 0.65, however, larger rerouting shares amplify both indirect NFLR and bridge-hub squeeze. Under δ=0.65, increasing r from 0.20 to 0.65 raises indirect peak NFLR from about 0.59% to 0.69% and bridge-hub squeeze from 5.3% to 8.1%. Rerouted traffic is therefore not an autonomous risk driver; it becomes consequential when shared hubs are already strongly coupled across geopolitical layers.

**Figure 8:**
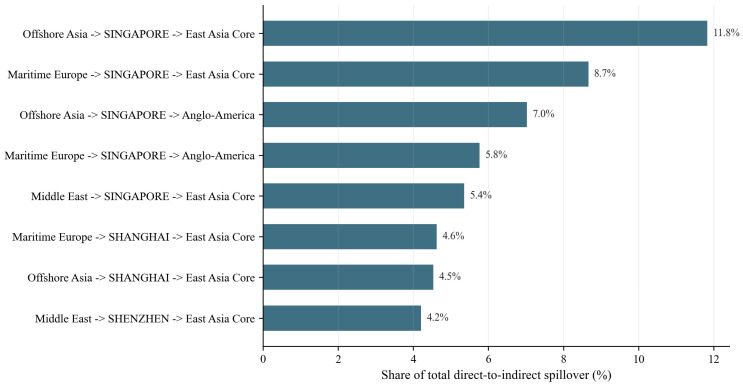
Dominant direct-to-indirect transmission paths under the baseline scenario.

Figure 8 makes this mechanism more explicit by tracing the dominant direct-to-indirect transmission paths. The strongest path is Offshore Asia → Singapore → East Asia Core, accounting for 11.8% of total direct-to-indirect spillover, followed by Maritime Europe → Singapore → East Asia Core (8.7%). This modeled path structure is consistent with the observed post-crisis pattern reported in recent Red Sea disruption studies, in which Europe–Asia services faced route restructuring, longer rotations, and stronger pressure on substitute hub systems. The model should therefore be read as an external-consistency stress test: it reproduces the expected direction of rerouting pressure toward shared transshipment interfaces, while not claiming to validate vessel-level canal passages or exact service-by-service diversions. Singapore clearly dominates the path structure, while Shanghai, Shenzhen, and Port Klang form a secondary bridge set. The main recipient of indirect pressure is East Asia Core, with a smaller branch transmitted toward Anglo-America. Spillover is therefore not distributed diffusely across the multiplex; it is funneled through a narrow set of bridge hubs and concentrated in a limited number of cross-layer corridors.

**Figure 9:**
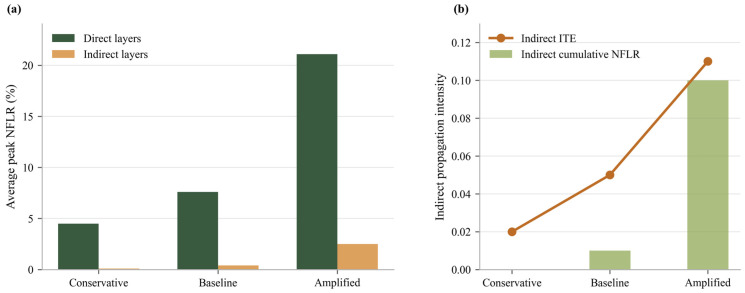
Regime-level amplification of direct and indirect propagation. Note: (**a**) average peak NFLR of direct and indirect layers under conservative, baseline, and amplified regimes; (**b**) indirect propagation intensity, showing indirect ITE and cumulative indirect NFLR across the three regimes.

Figure 9 summarizes these parameter effects across three interpretable regimes. Moving from conservative to amplified settings raises direct average peak NFLR from 4.5% to 21.1%, but it also raises indirect average peak NFLR from 0.1% to 2.5% and indirect ITE from 0.02 to 0.11. The key result is not only that stronger shocks cause larger direct losses but also that severe regimes convert a larger share of direct disruption into cross-layer propagation. Multiplexity therefore changes roles across regimes; it buffers local stress when spare capacity is sufficient, but it becomes a transmission structure when rerouted load and same-port coupling overwhelm shared hubs.

Taken together, the propagation results answer the third research question in dynamic terms. Cross-layer ties do not function as unconditional redundancy. They can absorb disruption when reserve capacity is sufficient, but they become channels of contagion when rerouted traffic is concentrated in shared hubs. Vulnerability is therefore shaped not only by where shocks originate but also by how spare capacity, hub concentration, and inter-layer overlap are jointly organized within the carrier–geopolitical multiplex.

### 4.4. Benchmark and Robustness Checks

The benchmark results indicate that the carrier–geopolitical layer structure is related to, but not reducible to, aggregate port size or carrier count. In the aggregated 2025 network, Singapore, Shanghai, and Shenzhen are the top three ports by endpoint occurrence, accounting for 3.52%, 3.30%, and 2.99% of all endpoint observations, respectively. Carrier-level overlap also identifies several broad multi-carrier hubs, including Shanghai and Changjiangkou, each served by 24 carriers. These results confirm that the main bridge ports are important in conventional aggregate and carrier-level representations. Thus, the benchmarks confirm that several bridge hubs are indeed important in conventional representations, but they also create a baseline against which the additional propagation role identified by the multiplex model can be evaluated.

However, the propagation results assign these ports a more specific role. Singapore accounts for 38.7% of cumulative direct-to-indirect spillover paths, followed by Shanghai (12.9%), Shenzhen (4.2%), and Port Klang (3.6%). Singapore, Shanghai, and Shenzhen are prominent in both aggregate and propagation-based views, but Port Klang provides a sharper validation case. It is not a top-five aggregate endpoint port, yet it appears as a secondary bridge hub and records the highest peak shared-hub squeeze share in the baseline scenario. This contrast shows that the multiplex model is not simply reproducing port-volume rankings; it identifies ports that mediate disruption between directly exposed and indirectly affected carrier–geopolitical layers.

**Figure 10:**
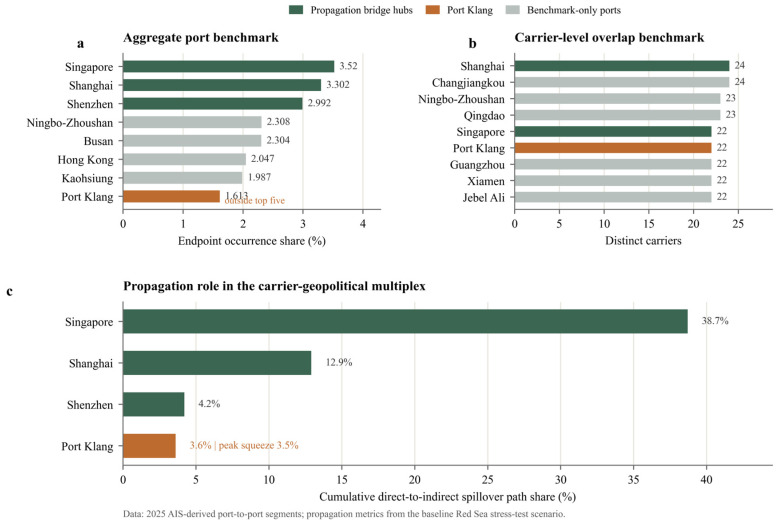
Benchmark comparison between aggregate port prominence, carrier-level overlap, and propagation role in the carrier-geopolitical multiplex. Note: (**a**) aggregate port benchmark based on endpoint occurrence share; (**b**) carrier-level overlap benchmark based on the number of distinct carriers; (**c**) propagation role in the carrier-geopolitical multiplex based on cumulative direct-to-indirect spillover path share.

Figure 10 further shows why the benchmark comparison matters for validating the proposed framework. Panel a shows that aggregate endpoint occurrence identifies Singapore, Shanghai, and Shenzhen as leading ports, but it places Port Klang outside the top five. Panel b shows that carrier-level overlap identifies a broader set of multi-carrier ports, including several benchmark-only ports that do not emerge as dominant propagation bridges. Panel c shows that the carrier–geopolitical multiplex isolates a narrower propagation role; Singapore dominates direct-to-indirect spillover, while Shanghai, Shenzhen, and Port Klang form a secondary bridge set. The divergence between panels indicates that bridge-hub importance in the propagation model cannot be fully inferred from aggregate port prominence or carrier count.

The permutation test provides a second check on the layer construction. The observed mean pairwise port Jaccard value across geopolitical layers is 0.1216, below all 500 random carrier-to-layer permutations preserving layer carrier counts, while the strongest observed pair remains Maritime Europe–Offshore Asia with a Jaccard value of 0.5317. This pattern suggests that the observed layer structure is not equivalent to a random partition of carriers with the same layer sizes. It therefore supports the interpretation that carrier–geopolitical affiliation captures differentiated network structure rather than merely relabeling carriers into arbitrary groups.

Taken together, the benchmark and permutation checks support the use of geopolitical affiliation as a strategic exposure layer. They show that the main findings are not reducible to aggregate port volume, carrier count, or random grouping, while leaving room for future comparisons with alliance-, ownership-, and service-agreement-based multiplexes.

## 5. Discussion

### 5.1. Multiplexity as Organizational Differentiation Rather than Mere Connectivity

The first implication of the results is conceptual. Once the GCLSN is reorganized as a carrier–geopolitical multiplex, connectivity is no longer a purely topological attribute of ports. It becomes an outcome jointly produced by organizationally differentiated carrier groups. This shift matters because the aggregate network can hide major differences in how connectivity is assembled. In the present case, layers vary sharply in size, cohesion, and dependence, which means that the same physical infrastructure supports different organizational logics at the same time. A port may appear central in the aggregate network while serving very different functions across carrier–geopolitical layers, and a layer may appear extensive without being internally well integrated.

This helps explain why intra-layer cohesion and inter-layer dependence do not move together mechanically. Maritime Europe and Offshore Asia occupy the structural core of the multiplex, but they do so through different internal configurations. Large scale and strong external overlap therefore do not necessarily imply balanced internal cohesion. Conversely, a smaller layer may appear internally cohesive while remaining weakly embedded in the wider system. The analytical implication is that multiplex prominence has at least two dimensions: internal organization and external entanglement. Treating the network as a single graph collapses these dimensions into one and obscures an important source of heterogeneity.

This is why Figure 2 is more than a descriptive scatterplot. It shows that Maritime Europe and Offshore Asia have similar external embeddedness but different internal cohesion, while Anglo-America and South America occupy the opposite position: high apparent cohesion but weak external dependence. The figure therefore underpins the paper’s central distinction between cohesion as an internal layer property and dependence as an inter-layer exposure property.

This point also speaks to a broader issue in shipping network research. Much of the existing literature treats firms as contributors to a shared route system but not as the basis of a differentiated multiplex structure. By assigning layers through carriers’ geopolitical affiliations, this study shows that organizational position is not a background characteristic; it is part of the network’s architecture itself. The liner system is therefore not just a transport network with geopolitical context around it. It is a geopolitical–organizational network whose transport structure is generated through partially overlapping carrier layers.

### 5.2. The Meaning of Interdependence

The second implication concerns the meaning of interdependence. The structural results show that overlap is concentrated rather than evenly distributed. A dense dependence core links Maritime Europe, Offshore Asia, the Middle East, and East Asia Core, while smaller layers are more asymmetrically embedded. This means that interdependence is not a universal property of the multiplex. Some layers are deeply entangled through repeated sharing of ports and routes, whereas others participate in the system through more selective attachments to the major layers.

The propagation results sharpen this interpretation by showing that structural overlap alone does not determine how risk moves. Secondary disruption does not spread across all shared ports equally. Instead, it is routed through a small set of bridge hubs, above all Singapore, and then through a secondary group including Shanghai, Shenzhen, and Port Klang. These ports are important not simply because they are large hubs in the conventional sense but because they function as interfaces between differently exposed carrier–geopolitical layers. In other words, the network’s dependence core and its propagation core overlap, but they are not identical. The most consequential nodes are those that connect direct-exposure layers to indirect-exposure layers under conditions of rerouting pressure. The benchmark checks reinforce this interpretation; aggregate port size and carrier count identify several important hubs, but they do not by themselves distinguish ports that convert direct corridor exposure into indirect cross-layer pressure.

This distinction is important for the interpretation of geopolitical vulnerability. If interdependence were diffuse, shocks would be expected to spread broadly and more evenly. What the model instead reveals is selective transmission through a narrow set of articulation points. The path analysis shows that the dominant corridors of secondary spillover are highly concentrated, especially toward East Asia Core through Singapore and then through East Asian shared hubs. The practical meaning of this pattern is that vulnerability is mediated by organizational interfaces, not just by corridor exposure in isolation. South America provides a useful boundary case for this argument. In Figure 2 it appears internally cohesive but externally peripheral, and Table 2 shows that it remains close to zero under the Red Sea baseline scenario. This does not imply that South American liner services are unimportant; rather, it shows that geographical distance and selective embedding reduce the probability that Red Sea pressure is transmitted into this layer through the observed shared-hub system. The result helps separate local cohesion from systemic exposure.

### 5.3. Rethinking Resilience in Carrier–Geopolitical Shipping Systems

The third implication concerns resilience. A common intuition in transport-network analysis is that more connections increase redundancy and therefore improve resilience. The present results show that this intuition is incomplete in a multiplex organizational system. Cross-layer ties can indeed absorb shocks under some conditions, but they can also transmit them. Whether multiplexity buffers or amplifies disruption depends on how spare capacity, rerouting opportunities, and overlap are distributed across layers and hubs.

This is where the load–capacity framework becomes especially useful. Figure 6 indicates that indirect disruption remains limited across much of the alpha–delta parameter range and rises sharply only when weak tolerance coincides with strong inter-layer squeeze. The delta–reroute phase space in Figure 7 adds a second condition: rerouting becomes consequential only when the shared-hub system is already strongly coupled. Taken together, these results imply that vulnerability in the multiplex is nonlinear. It does not increase smoothly with connectivity. Instead, it intensifies once concentrated bridge hubs face the combined effects of reduced reserve capacity, stronger cross-layer pressure, and detoured traffic.

This also reframes the meaning of resilience at the carrier level. A carrier or layer may appear diversified because it participates in many ports and routes, yet that diversification may still be organized through the same limited set of shared hubs as other layers. Under those conditions, apparent redundancy is partly illusory. The relevant question is not simply whether alternative links exist, but whether those alternatives are distributed across distinct capacity pools or converge on the same chokepoints. In this sense, resilience in the carrier–geopolitical multiplex is better understood as distributed flexibility rather than as connection abundance alone.

More broadly, the discussion suggests that the global liner system should be interpreted as a complex organizational infrastructure whose vulnerability is shaped by heterogeneous layer structures, asymmetric dependence, and selective cross-layer propagation. From this perspective, the central issue is no longer whether the network is connected, but how connectivity is partitioned, shared, and stressed across carrier–geopolitical structures. That shift in perspective is the main conceptual value of the study.

## 6. Conclusions

### 6.1. Main Findings

This paper examined GCLSNs as a carrier–geopolitical multiplex rather than as a single aggregated transport graph. Using vessel records from Clarksons and AIS-derived voyage sequences for 2023–2025, it reconstructed yearly weighted multiplex networks in which layers are defined by carriers’ geopolitical affiliations. On that basis, the analysis addressed three questions concerning structural differentiation, inter-layer dependence, and the dynamic propagation of geopolitical disruption.

Three main findings follow. First, carrier–geopolitical layers differ substantially in internal structure. Layer size, efficiency, centralization, and cohesion do not coincide mechanically, which means that aggregate connectivity conceals major differences in how liner networks are organized. Second, inter-layer dependence is strong but uneven. The multiplex contains a dense dependence core among the major layers, while smaller layers are embedded more asymmetrically in larger carrier–geopolitical structures. Third, multiplexity does not imply resilience by itself. Under the Red Sea load–capacity simulations, cross-layer ties can buffer disruption when reserve capacity is sufficient, but they become channels of transmission when rerouted traffic is concentrated at strongly coupled bridge hubs.

The path and sensitivity analyses reinforce this conclusion. Secondary disruption is transmitted through a limited number of shared articulation hubs, especially Singapore and then a smaller set including Shanghai, Shenzhen, and Port Klang. At the same time, nonlinear amplification emerges only under specific combinations of low tolerance, strong inter-layer squeeze, and elevated rerouting intensity. Vulnerability in the global liner system is therefore shaped not only by where shocks originate but also by how organizational dependence and spare capacity are distributed across the multiplex network.

### 6.2. Implications, Limitations, and Future Research

The study has several implications. For shipping network analysis, it shows the value of moving from aggregate topology toward a carrier-affiliated multiplex structure. For resilience research, it suggests that redundancy should not be inferred from connection density alone; what matters is whether alternative connectivity is distributed across distinct capacity pools or concentrated in the same bridge hubs. For maritime governance and carrier strategy, the results imply that crisis planning should pay particular attention to shared hubs that connect directly exposed and indirectly exposed layers, since these are the main interfaces through which secondary disruption is propagated.

At the same time, the study has clear limitations. Geopolitical affiliation is assigned at the carrier level through headquarters and principal strategic registration, which are analytically tractable but cannot capture every nuance of alliance membership, vessel-sharing agreements, ownership structure, subsidiaries, chartering relationships, or state influence. The load–capacity model is designed as a comparative stress test rather than a calibrated operational forecast, so its primary value lies in revealing relative propagation mechanisms rather than exact disruption magnitudes. In addition, the Red Sea scenario is also based on an observable AIS port-call proxy for corridor exposure, which may underestimate services exposed through canal transit or long-distance route rotations without corridor-port calls.

These limitations point to several directions for future research. First, alternative multiplex definitions based on alliance-level coordination, ownership links, vessel-sharing agreements, or regional service structures could be compared with the present carrier–geopolitical layer construction. Second, the load–capacity framework could be calibrated with richer operational data, including port-level throughput, berth and yard utilization, vessel schedule reliability, transshipment dwell time, and observed post-crisis service adjustments. Third, additional geopolitical scenarios beyond the Red Sea could be used to test whether different crisis types activate the same shared hubs or shift the location of principal propagation pathways. Such extensions would help clarify how geopolitical fragmentation, operational capacity, and network complexity jointly reshape the resilience of global maritime logistics.

## Figures and Tables

**Table 1:** Interpretation of parameters in the load–capacity stress-test model.

Parameter	Role in the Model	Interpretation in this Study	Calibration Status
Tolerance coefficient α	Capacity margin above pre-shock load	Spare capacity under different stress-test regimes	Varied across conservative, baseline, amplified, and sensitivity settings
Capacity nonlinearity η	Scaling of reserve capacity with node load	Whether high-load ports receive proportionally more or less reserve capacity	Sensitivity parameter
Redistribution preference μ	Allocation of excess load to active neighbors	Whether redistribution favors strong routes or residual capacity	Sensitivity parameter
Rerouting share r	Share of excess load redirected to substitute hubs	Scenario-based detour pressure from disrupted corridor nodes	Varied in one-at-a-time and phase-diagram tests
Spillover intensity δ	Strength of cross-layer resource squeeze	How pressure at shared hubs reduces effective capacity in other layers	Sensitivity parameter
Shock intensity s	Initial capacity reduction at exposed corridor nodes	Severity of the Red Sea stress scenario	Scenario parameter

**Table 2:** Intra-layer structure of carrier–geopolitical subnetworks in 2025.

Layer	Density (%)	Clustering	Centralization	Efficiency	Cohesion Index
Maritime Europe	2.022	0.606	0.610	1.494	0.153
Offshore Asia	1.718	0.589	0.617	1.141	−0.219
East Asia Core	3.056	0.601	0.582	1.066	−0.086
Middle East	2.430	0.544	0.524	0.764	−0.345
Gateway States	5.421	0.551	0.507	0.724	−0.195
Anglo-America	13.500	0.561	0.423	0.742	0.410
South America	28.889	0.767	0.315	0.925	2.069

**Table 3:** Layer asymmetry under the baseline Red Sea load–capacity scenario in 2025.

Layer	Status	Corridor Exposure (%)	Peak NFLR (%)	Cumulative NFLR	Time to Peak	Peak Failed-Node Share (%)	ITE from Direct Core
Maritime Europe and the Maghreb	Direct	3.2	9.4	0.31	3	2.0	1.17
Gateway States	Direct	6.9	9.0	0.34	3	8.0	1.26
Offshore Asia and Oceania	Direct	2.7	6.2	0.21	2	2.7	0.79
The Middle East Shatterbelt	Direct	3.8	5.7	0.21	3	2.3	0.78
East Asia—Continental Core	Indirect	0.0	1.0	0.03	3	0.4	0.11
Anglo-America and the Caribbean	Indirect	0.0	0.3	0.01	3	0.0	0.03
South America	Indirect	0.0	0.0	0.00	2	0.0	0.00

## Data Availability

The data used in this study are derived from Clarksons vessel information and AIS-based vessel movement records. Due to licensing restrictions and the commercial sensitivity of the raw data, the complete underlying dataset cannot be made publicly available. Processed data supporting the main empirical results may be made available from the corresponding author upon reasonable request for non-commercial research purposes.

## Supplementary Materials

The following supporting information can be downloaded at: https://www.mdpi.com/article/10.3390/e28070723/s1, Figure S1: Interactive multiplex network visualization.

## Appendix A. Carrier–Geopolitical Affiliation Scheme

Table A1 reports the carrier–geopolitical affiliation scheme used to construct the multiplex layers. Carrier market shares are based on the Clarksons ranking used for sample selection. Geopolitical layers are assigned according to Cohen’s geopolitical regionalization and the location of each carrier’s headquarters or principal strategic registration.

**Table A1:** Carrier–geopolitical affiliation scheme.

Clarksons Rank	Carrier	Market Share (%)	Sample Cumulative Share (%)	Headquarters/Registration	Cohen Geopolitical Layer
1	MSC	20.2	20.2	Geneva/Sorrento, Switzerland/Italy	Maritime Europe and the Maghreb
2	Maersk	14.3	34.5	Copenhagen, Denmar	Maritime Europe and the Maghreb
3	CMA CGM	12.7	47.2	Marseille, France	Maritime Europe and the Maghreb
4	COSCO Shipping Lines	10.6	57.8	Shanghai, China	East Asia—Continental Core
5	Hapag-Lloyd	7.4	65.2	Hamburg, Germany	Maritime Europe and the Maghreb
6	ONE	6.2	71.4	Tokyo, Japan	Offshore Asia and Oceania
7	Evergreen Marine	5.7	77.1	Taoyuan City, Taiwan	Offshore Asia and Oceania
8	HMM	2.9	80.0	Seoul, South Korea	Offshore Asia and Oceania
9	ZIM Integrated Shpg	2.5	82.5	Haifa, Israel	The Middle East Shatterbelt
10	Yang Ming	2.2	84.7	Keelung, Taiwan	Offshore Asia and Oceania
11	Wan Hai Lines	1.7	86.4	Taipei, Taiwan	Offshore Asia and Oceania
12	PIL	1.0	87.4	Singapore, Singapore	Offshore Asia and Oceania
13	X-Press Feeders	0.6	88.0	Singapore, Singapore	Offshore Asia and Oceania
14	SITC	0.5	88.5	Hong Kong, Hong Kong	East Asia—Continental Core
15	Unifeeder	0.5	89.0	Aarhus, Denmark	Maritime Europe and the Maghreb
16	KMTC	0.5	89.5	Seoul, South Korea	Offshore Asia and Oceania
17	IRISL	0.5	90.0	Tehran, Iran	The Middle East Shatterbelt
18	CSAV Austral SPA	0.5	90.5	Santiago, Chile	South America
19	T.S. Lines	0.5	91.0	Taipei, Taiwan	Offshore Asia and Oceania
20	Matson Inc	0.4	91.4	Honolulu, United States	Anglo-America and the Caribbean
21	Arkas Line	0.4	91.8	Izmir, Turkey	Gateway States
22	Swire Shipping	0.3	92.1	Hong Kong, Hong Kong	East Asia—Continental Core
23	SeaLead Shpg	0.3	92.4	Singapore, Singapore	Offshore Asia and Oceania
24	Sinokor Merchant	0.3	92.7	Seoul, South Korea	Offshore Asia and Oceania
25	Zhonggu Logistics	0.3	93.0	Shanghai, China	East Asia—Continental Core
26	Emirates	0.3	93.3	Dubai, United Arab Emirates	The Middle East Shatterbelt
30	Global Feeder Shpg	0.2	94.1	Singapore, Singapore	Offshore Asia and Oceania

Note: Clarksons rank reports the original carrier ranking used for sample selection. Skipped rank numbers indicate carriers that were not retained in the final vessel-matched analytical sample; therefore, the jump from rank 26 to rank 30 does not indicate a missing table row.
